# Interaction of Opioids with TLR4—Mechanisms and Ramifications

**DOI:** 10.3390/cancers13215274

**Published:** 2021-10-21

**Authors:** Mai Mahmoud Gabr, Iqira Saeed, Jared A. Miles, Benjamin P. Ross, Paul Nicholas Shaw, Markus W. Hollmann, Marie-Odile Parat

**Affiliations:** 1 School of Pharmacy, The University of Queensland, St. Lucia, QLD 4072, Australia; m.gabr@uq.edu.au (M.M.G.); iqira.saeed@uq.edu.au (I.S.); j.miles1@uq.edu.au (J.A.M.); bross1@uq.edu.au (B.P.R.); n.shaw@uq.edu.au (P.N.S.); 2 Academic Medical Center, Department of Anaesthesiology, 1100DD Amsterdam, The Netherlands; m.w.hollmann@amsterdamumc.nl

**Keywords:** toll-like receptor 4, morphine, opioids, lipopolysaccharide

## Abstract

**Simple Summary:**

Recent evidence indicates that opioids can be active at a receptor that is abundantly expressed on innate immune cells as well as cancer cells: the receptor is termed toll-like receptor 4 (TLR4). TLR4 is increasingly recognised as playing key roles in tumour biology and anticancer defences. However, the issue of whether TLR4 mediates some of the effects of opioids on tumour growth and metastasis is entirely unknown. We review existing evidence, mechanisms, and functional consequences of the action of opioids at TLR4. This opens new avenues of research on the role of opioids in cancer.

**Abstract:**

The innate immune receptor toll-like receptor 4 (TLR4) is known as a sensor for the gram-negative bacterial cell wall component lipopolysaccharide (LPS). TLR4 activation leads to a strong pro-inflammatory response in macrophages; however, it is also recognised to play a key role in cancer. Recent studies of the opioid receptor (OR)-independent actions of opioids have identified that TLR4 can respond to opioids. Opioids are reported to weakly activate TLR4, but to significantly inhibit LPS-induced TLR4 activation. The action of opioids at TLR4 is suggested to be non-stereoselective, this is because OR-inactive (+)-isomers of opioids have been shown to activate or to inhibit TLR4 signalling, although there is some controversy in the literature. While some opioids can bind to the lipopolysaccharide (LPS)-binding cleft of the Myeloid Differentiation factor 2 (MD-2) co-receptor, pharmacological characterisation of the inhibition of opioids on LPS activation of TLR4 indicates a noncompetitive mechanism. In addition to a direct interaction at the receptor, opioids affect NF-κB activation downstream of both TLR4 and opioid receptors and modulate TLR4 expression, leading to a range of in vivo outcomes. Here, we review the literature reporting the activity of opioids at TLR4, its proposed mechanism(s), and the complex functional consequences of this interaction.

## 1. Introduction

**Figure 1:**
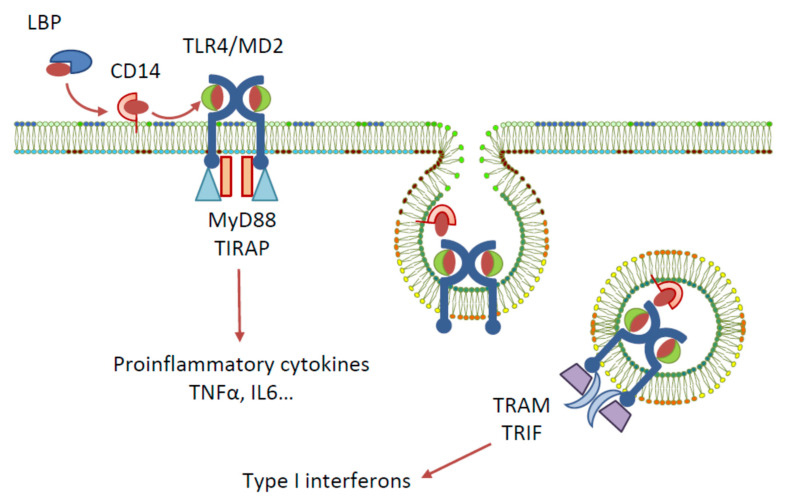
Signalling pathways elicited by LPS activation of TLR4.

Toll-like receptors (TLRs) are a family of pattern recognition receptors first identified in Drosophila, and to date, ten members of the TLR family are recognised in humans [1]. One of the most widely studied TLRs is TLR4, a membrane-bound receptor that has an extracellular leucine-rich repeat domain, a transmembrane domain, and a cytoplasmic toll/interleukin-1 receptor (TIR) domain [2]. Among other pattern recognition receptors, TLR4 plays a role in the innate immune system by recognising various pathogen-associated molecular patterns (PAMPs) and damage-associated molecular patterns (DAMPs), and by triggering host defences to produce inflammatory cytokines, eliminating the cause of danger [3]. The activation of TLR4 by natural ligand lipopolysaccharide (LPS)—a PAMP contained in gram-negative bacteria cell membranes—requires the involvement of the following accessory proteins: lipopolysaccharide binding protein (LBP), the cluster of differentiation antigen 14 (CD14), and myeloid differentiation protein 2 (MD-2) [4] (Figure 1). Signal transduction is initiated by the binding of LPS to LBP, which traffics it to CD14. CD14 then binds to the cell and delivers LPS to the TLR4-associated MD-2. The detection of LPS by TLR4 causes the dimerisation of the TLR4 cytoplasmic TIR domain, which can then recruit different sets of adaptor proteins and initiate two downstream signalling pathways: the MyD88- and TRIF/TRAM-dependent pathways. Triggering of the MyD88-dependent signalling pathway activates the transcription factors NF-κB, IRF5, AP-1, and CREB, and the subsequent production of pro-inflammatory cytokines. The endocytosis of the TLR4 complex, however, recruits the TRIF/TRAM pathway, resulting in the activation of IRF-3 and the production of type I interferons, or, alternatively, the activation of NF-κB via RIP1 kinase interactions with TRAF6 and the production of cytokines [5,6].

TLR4 is increasingly recognized as playing important roles in cancer (reviewed in [7,8]). It is abundantly expressed on tumoural immune and non-immune stromal cells and is often overexpressed by tumour cells [7,9]. TLR4 activation of cancer cells is mostly reported to promote aggressive behaviour of cancer cells, including epithelial to mesenchymal transition, migration, and invasion [10,11,12,13,14]; furthermore, TLR4 overexpression has been correlated with increased metastasis (reviewed in [8]). TLR4 activation in the tumour microenvironment further maintains a tumour-favourable inflammatory response [15] and DAMPs expressed by cancer cells can promote angiogenesis [16]. A major endogenous TLR4 agonist, that is relevant to cancer, is the DAMP high-mobility group box 1 (HMGB1), which possesses protumour characteristics through sustaining an anti-inflammatory environment and promoting invasion metastasis and angiogenesis [15]. HMGB1 that is produced by tumour cells interacts with TLR4 on platelets, causing their activation, adhesion, and release of pro-metastatic factors, resulting in metastasis in mice [17]. On the other hand, it has also been documented that TLR4 activation on immune cells is protective in the context of cancer [18] and is necessary for the efficacy of chemotherapy or radiotherapy [19]. Treatment with a synthetic TLR4 agonist increased innate and adaptive immunity and led to reduced metastasis in several rodent models [20]. An elegant study has dissected out a pathway—essential in the study of anti-cancer immunity in mice and humans—whereby HMGB1 that is secreted by dying tumour cells activates TLR4 on dendritic cells. Functional TLR4, and the adaptor MyD88, are required for dendritic cells to cross-present antigens from the dying tumour cells and activate tumour-specific T-cell immune response [19]. TLR4 therefore plays a dual role in cancer.

Contemporary literature indicates that opioids can be active at TLR4; however, whether this contributes to the action of opioids on tumour growth and metastasis is, to date, entirely unexplored. We reviewed existing evidence, mechanisms, and functional consequences of the action of opioids at TLR4.

## 2. Opioids Inhibit LPS-Induced Activation

Evidence of a possible in vitro connection between opioids and TLR4 originates from studies that examined the effects of LPS, the classical TLR4 agonist, on cultured primary brain cells, as well as the ability of opioids to inhibit these effects [21,22,23,24]. Das et al. reported a concentration-dependent increase in the secretion of IL-lβ, upon treating the mixed brain cell cultures of embryonic mice with either LPS or with the endogenous opioid peptide [Met ^5^]-enkephalin [21]. These effects were partially inhibited by naloxone (10^−9^ M–10^−6^ M) in a concentration-dependent fashion. Additionally, LPS induced morphological changes to the microglia in the brain cultures, which were blocked upon prior treatment with naloxone. Similar findings were reported by Kong et al., where the LPS-induced pro-inflammatory effects in mouse primary mixed glial cultures were significantly inhibited by naloxone, as well as by the endogenous opioid peptide dynorphin (dyn) A-(1–8) [22]. Naloxone (10^−10^–10^−6^ M) caused a concentration-dependent inhibition of LPS effects, reaching maxima of 55% and 21% inhibitions of NO and TNF-α production, respectively. The suppression of LPS effects by ultra-low dynorphin concentrations (10^−16^–10^−12^ M) was also observed, resulting in inhibitions of up to 29%, 39%, 32%, and 25% of the LPS-induced secretions of NO, TNF-α, IL-1α, and IL-6, respectively. Naloxone was later shown to prevent the effects of LPS in BV-2 microglial cells [25]. Further studies employed immune cells to evaluate the effect of opioids on LPS-induced activation. At very high concentrations (1 mM), morphine was reported to inhibit LPS-induced lymphocyte proliferation in a naloxone-insensitive fashion; however, this study did not demonstrate whether the cells were still viable at such concentrations [26]. Also at elevated concentrations (10^−5^–10^−6^ M), deltorphin-D_variant_—the δ_2_-specific opioid receptor agonist—was documented to suppress LPS-induced MAPK activation and the expression of TNF and MIP-2 in RAW264.7 macrophages [27]. Remifentanil (but not the structurally related compounds fentanyl, sufentanil, or alfentanil) was able to attenuate LPS-induced activation of neutrophils in vitro [28]. In this study, however, the effect of remifentanil was reversed by a κ-opioid receptor antagonist. Interestingly, the abilities of mM concentrations of morphine, tramadol, or ketobemidone to prevent the LPS-induced release of TNF-α and IL-8 by U-937 cells in vitro was facilitated, rather than countered, by naloxone [29]. In vitro studies of opioids modifying the effects of LPS are summarised in Table 1. It is noteworthy that opioids with heterogenous structural features can interfere with LPS-induced activation, which may indicate a variety of levels of action. Moreover, inhibition of the effects of LPS could not be correlated with opioid receptor subtype, since μ (e.g., ketobemidone, fentanyl), κ (e.g., salvinorin A, U50488), δ (e.g., deltorphine, TAN-67)-selective agonists, or non-selective agonists (e.g., oxycodone) all inhibited the effects of LPS. The observation that opioid agonists, antagonists, or endogenous opioids prevented the effects of LPS was verified in a number of in vivo models; however, the in vivo setting could denote an indirect effect, and this was not interpreted to mean that opioids interfere directly with TLR4 activation. For example:

○ Morphine (1 mg/L) prevented waterborne LPS-induced signalling in zebrafish embryos [30], although lower concentrations (100 ng/L or 100 μg/L) had the opposite effect and exacerbated LPS-induced inflammation;
○ Morphine prevented LPS-induced synovial inflammation when both were injected intra-articularly in horses [31];
○ Naltrexone prevented iNOS induction in splenocytes, and NO production in the circulation of rats injected intraperitoneally with LPS [32], although the possibility that this effect might be medicated via TLR4 activity was not evoked—instead, the involvement of CNS endogenous opioids was suggested, since the peripheral opioid antagonist N-methyl naltrexone was ineffective in counteracting LPS unless injected intrathecally;
○ Endomorphin-1, administered intravenously, was protective in a mouse model of lung injury and inflammation following intratracheal instillation of LPS [33];
○ The μ opioid receptor antagonist β-funaltrexamine could prevent LPS-induced neuroinflammation in mice [34].

When viewed in combination, these results indicate that both opioid agonists and antagonists can prevent the effects of LPS in cultured cells or in live animals.

**Table 1:** In vitro studies testing the effect of opioids on LPS-induced activation.

Cells	Opioid Agent	Concentration	Readout of LPS-Induced TLR4 Activation	Impact of Opioid Agent on LPS Effect	Reference
Rat splenocytes	α- and β-endorphin	3.3–330 nM	Splenocyte proliferation	No effect	[4]
Mouse embryonic mixed brain cell cultures	Naloxone	10^−9^ M–10^−6^ M	Increase in production of IL-lβ Morphological changes to the microglia	Concentration-dependent inhibition	[21]
Mouse primary mixed glial cultures	Naloxone Dynorphin (dyn) A-(1–8)	10^−10^–10^−6^ M 10^−16^–10^−12^ M	Production of NO, TNF-α, IL-1α, and IL-6.	Inhibition	[22]
Rat mesencephalic neuron–glia cultures Microglia-enriched cultures	(+) and (−)-Naloxone	1 µM	Microglial activation (nitrite accumulation, rise in TNF-α and IL-1β levels) Inflammatory damage (reduction in high affinity dopamine uptake and decrease in number of healthy neurons) Superoxide generation	Significant reduction	[23]
Mouse primary cortical neuron–glia co-cultures	(+) and (−)-Naloxone	1 µM	Production of TNF-α and NO Inflammatory damage (Morphological changes, decrease in number of healthy neurons and increased release of LDH)	Significant attenuation	[24]
RAW264.7 mouse macrophages	δ_2_-specific agonist Deltorphin D_variant_	10^−5^ to 10^−6^ M	TNFα and MIP-2 production	Significant attenuation via suppression of p38 MAPK activation	[27]
Human dendritic cells	Morphine	10^−8^ to 10^−12^ M	Dendritic cell maturation (upregulation of HLA-DR, CD86 CD80 CD83)	Augmentation	[35]
Whole blood	Remifentanil Fentanyl	1–100 ng/mL 2–200 ng/mL	Cytokine release: TNFα, IL-10 IL-6	Inhibition	[36]
BV2 microglial cells	Naloxone	1 μM	Expression of HSP60, HSF-1, NF-κB, iNOS, TNF-α, IL-1β, IL-6, and nitric oxide	Significant inhibition	[37]
HEK-Blue™ hTLR4 cells	(+) and (−)-Naloxone (+) and (−)-Naltrexone	10 μM 10 μM	Iincrease in SEAP expression	Non-stereoselective, noncompetitive inhibition	[38]
Rat microglial cells (HAPI)	(+)-Naloxone	1 μM	Rrelease of IL-1, CD11b, and IL-6 mRNA	Significant attenuation	
RAW264.7 mouse macrophages	(+) and (−)-Naloxone		Cytosol to cell membrane clearance of the green fluorescent protein-tagged Akt1	Significant attenuation	[39]
HEK-Blue™ hTLR4 cells	Morphine Fentanyl Naltrexone β-FNA	3–100 µM 1–100 µM 30–1000 µM 3–30 µM	Increase in SEAP expression	Significant inhibition	[40]
Bone marrow derived mast cells	Morphine	0.1 μM–1 mM	TNF exocytosis	Inhibition	[41]
U-937 human monocytic cells	Morphine Fentanyl Tramadol Ketobemidone	0.0015–1.5 mM 0.002–2.3 μM 0.0042–4.2 mM 0.00175–1.75 mM	Increase in TNF-α and IL-8	High concentration of morphine tramadol and ketobemidone prevents TNF-α, high concentration of tramadol and ketobemidone prevents IL-8 induction	[29]
Human neutrophils	Remifentanil Alfentanil Sufentanil Fentanyl	1–100 ng/mL 10–1000 ng/mL 1–100 ng/mL 1–100 ng/mL	Increase in TNF-α, IL-6 and MAPK activation	Only remifentanil attenuated the effects of LPS	[28]
HEK-Blue™ hTLR4 cells	(+) and (−)-Naloxone (+) and (−)-Naltrexone	0.01–100 μM	Increase in SEAP expression	No effect	[42]
BV-2 microglial cells	(+)-Naltrexone	50–400 µM	NO production	Concentration-dependent inhibition	[43]
Bone marrow derived macrophages	Morphine	1 μM	Autophagy LPS-induced autophagolysosomal fusion	Facilitation Inhibition	[44]
Mouse primary microglial cells	Morphine	100 nm and 10 μM	NF-κB signalling Unstimulated NF-κB signalling	Potentiation No effect	[45]
THP-1 human macrophage cell line	Dynorphin N-terminal fragments	10 nM	Nuclear factor-κB translocation, IL-1β and TNF-α release	Reduction	[46]
Wistar rat primary microglial cultures	Biphalin	0.1, 1, 10, 20 μM 10 μM	NO production Increase of Iba1, phosphorylation of nuclear factor-κB, iNOS, IL-1β, IL-18, COX-2, NLRP3, IL-6, IL-10, TNF-α, and TRIF	Significant attenuation (except for 20 μM Biphalin) Significant attenuation	[47]
BV-2 mouse microglial cells	Naltrexone and Naloxone (+)-Naltrexone and (+)-Naloxone (+)-Naltrexone and (+)-Naloxone (+)-Naltrexone and (+)-Naloxone	1–400 μM 10–400 μM 20–100 μM 5–200 μM	NO production ROS production and phagocytosis TNF-α production IL-1β production	Non-stereoselective concentration-dependent inhibition Concentration-dependent inhibition Concentration-dependent inhibition No effect	[48]
Rat primary neonatal microglia and rat primary peritoneal macrophages	(+)-Naltrexone and (+)-Naloxone (+)-Naltrexone and (+)-Naloxone	1–400 μM 100–400 μM	NO production TNF-α production	Concentration-dependent inhibition Concentration-dependent inhibition	
HEK-Blue™ hTLR4 cells	Morphine M3G	1–100 µM 1–100 µM	Increase in SEAP expression	Significant inhibition	[49]
Mouse primary cortical astrocytes	Naloxone Nalmefene Naloxone Nalmefene	5–300 µM 0.01–1 µM 150 µM 0.1 µM	Superoxide generation NF-κB activation and iNOS production	Concentration-dependent inhibition Significant inhibition	[50]
Peripheral blood mononuclear cells (PBMC) and isolated CD14+ monocytes from PBMC	Naltrexone	1–200 μM	Production of IL-6 and TNF-α	No effect	[51]
THP-1 human macrophage cell line	Dynorphin 3–14	10 nM and 1 µM 10^−17^–10^−11^ M 10^−11^–10^−7^ M	NF-κB/p65 nuclear translocation Release of IL-1β Release of TNF-α	Significant inhibition Significant inhibition Significant augmentation	[52]
HEK-Blue™ hTLR4 cells	Dynorphin 3–14	10^−8^–10^−5^ M	Increase in SEAP expression	Concentration dependent inhibition	
H9C2 cardiomyocytes	Remifentanil	2.5 μM	Cell death, LDH release, MDA production, PKCβ activation, autophagy, and decrease SOD activity	Attenuation	[53]
Human aortic endothelial cells	Remifentanil	2.5 μM	Superoxide production iNOS ICAM-1 PARP1 expression, NF-κb activation	Attenuation	[54]
Primary hippocampal astrocytes	Oxycodone	5–20 μg/mL	NF-κb activation and expression of TNF-α, IL-6, and IL-1β	Inhibition	[55]
NR8383 Macrophages	κOR agonists salvinorin A and U50488	10^−9^–10^−11^ M	Nitrosative stress, TNF-α, IL-1β, iNOS, and COX2 expression	Inhibition	[56]
RAW264.7 murine macrophages	Naltrexone	5 μM	IL-1β, IL-6, and mcp-1 and ERK1/2 phosphorylation	Inhibition	[57]
Bovine endometrial cells	β-endorphin	20–200 pg/mL	NF-κb activation and expression of TNF-α, IL-6, and IL-1β iNOS	Inhibition	[58]
Human pulmonary microvascular endothelial cells	Oxycodone	23 μM	Permeability, apoptosis, TNF-α, IL-1β, and MMP9 caspase-3 expression	Inhibition	[59]
BV2 microglial cells	TAN-67 δ-opioid agonist	3–10 μM	Cell death, IL-1β IL-6 and iNOS expression, MAPK singnalling	Inhibition	[60]
C6 glial cells	((D-Arg^2^, Lys^4^)-Dermorphin-(1–4)-amide) DALDA	10–20 μM	Nitrosative stress	Inhibition	[61]
Mouse bone marrow-derived macrophages	Butorphanol	8 μM	Expression levels of iNOS, IL-6, TNF-a, and IL-1b in M1-polarized BMDMs	Inhibition	[62]

## 3. Opioid Receptor Active and Inactive Isomers Both Inhibit LPS-Induced Activation

The non-stereoselective effect of opioids at TLR4 refers to the ability of (+) opioid isomers to interact with TLR4, while opioid receptors are selective for (−) isomers. The inhibitory effects of naloxone isomers on microglial activation, and the inflammatory damage of dopaminergic neurons induced by LPS, have been reported [23]. Treatment of rat mesencephalic neuron–glia cultures with LPS (0.1–100 ng/mL) induced microglial activation, as evidenced by nitrite accumulation and a rise in levels of the pro-inflammatory cytokines TNF-α and IL-1β, and resulted in inflammatory damage, as reflected by a reduction in high affinity dopamine uptake and a decrease in the number of healthy neurons. These effects were significantly reduced upon pre-treatment by 1 µM (−)- or (+)-naloxone, with equal potencies for both stereoisomers. Furthermore, naloxone caused a significant non-stereospecific, concentration-dependent reduction of the superoxide generation that was induced by LPS in mixed neuron–glia cultures and microglia-enriched cultures and was also proven to interfere with LPS binding to cell surface receptors. The same group reported similar findings in mouse primary cortical neuron–glia co-cultures [24], and the results were further confirmed by a preclinical study, where both (−) and (+)-naloxone inhibited the activation of microglia after LPS injection in the *substantia nigra* of rats [63]. However, toll-like receptors were not directly mentioned in this report, since opioid receptors only respond to (−)-opioid stereoisomers, the involvement of non-opioid receptors was proposed based on the similarities in inhibition observed for both (−) and (+)-opioid isomers in a number of studies (Table 1). More recently, (+)-naloxone was shown to prevent the effects of LPS in vivo in a model of inflammation-induced pre-term birth in mice [64].

## 4. Opioids Exert Agonistic and Antagonistic Effects at TLR4

The atypical, non-stereoselective effects of opioids continued to be unexplained until a link to TLR4 was proposed, with several reports on the effects of both opioid agonists and antagonists using different cell types, as well as in TLR4-specific reporter cell lines. Hutchinson et al. were the first group to report the blockade of LPS-induced effects by opioid antagonists in HEK-Blue™ hTLR4 cells and in rat microglial cells (HAPI) [38]. HEK-Blue™ hTLR4 cells are engineered human embryonic kidney 293 (HEK 293) cells. These cells are stably transfected to overexpress human TLR4, its accessory proteins MD-2 and CD14, and a nuclear factor kappa B (NF-κB)-inducible reporter gene (namely, secreted embryonic alkaline phosphatase, SEAP) [65]. LPS (0.01–100 ng/mL) increased SEAP expression in HEK-Blue™ hTLR4 cells, and this was significantly inhibited by 10 μM naloxone or naltrexone. The antagonism of TLR4 activation by naloxone and naltrexone was non-stereoselective and, unlike the competitive antagonistic effect of lipopolysaccharide from the photosynthetic bacterium *Rhodobacter sphaeroides* (LPS-RS), the effect of opioids was non-competitive in nature as indicated by the inhibition curves (i.e., the magnitude of the maximum attained response to LPS was reduced) [38]. Similar observations were reported in the same study, using the rat microglia cell line HAPI, where 1 μM (+)-naloxone significantly attenuated the LPS-induced activation of microglia, as measured by expression of IL-1, CD11b, and IL-6 mRNA. These results implied the blockade of TLR4 function by opioid antagonists, which could occur at the receptor level or at other downstream signalling sites.

Subsequently, RAW264.7 mouse macrophages were employed in a study to examine opioid effects on TLR4 signalling [39]. The activation of TLR4 triggers three main intracellular pathways, NF-κB, MAPK, and PI3k/Akt1. While NF-κB and MAPK are associated with the induction of pro-inflammatory responses, PI3k is involved in cell survival and motility. RAW264.7 cells expressing a green fluorescent protein (GFP)-tagged Akt1, show a diffuse green fluorescence in the cytosol under basal conditions, which is rapidly translocated to the plasma membrane upon activation. Robust and rapid cytosol-to-cell membrane clearance of the GFP-tagged Akt1 was induced by LPS; this effect was significantly attenuated upon pre-incubation with LPS-RS, (+)-naloxone, or (−)-naloxone. However, LPS-RS, (+)-naloxone, and (−)-naloxone did not prevent cytosol-to-cell membrane clearance when Akt1 was activated via a TLR4-independent pathway. These observations strongly linked the naloxone blocking effects to its interference with the TLR4 signalling pathway [39], either at the LPS binding level or through the TLR4 recruitment of adapters TRIF/TRAM.

In addition to the inhibition of LPS-induced signalling, a separate study showed that 200 µM (+)- and (−)-morphine, per se, produced a significant, prolonged GFP-Akt1 cytosolic clearance, suggesting that opioids can activate TLR4 signalling [39]. This effect was significantly inhibited by LPS-RS (200 ng/mL), (+)-naloxone (200 µM), and (−)-naloxone (200 µM). The ability of opioid agonists to activate TLR4 was confirmed using HEK-Blue™ hTLR4 cells, where (+)-morphine, (−)-morphine, and the opioid receptor-inactive metabolite morphine-3-glucuronide (M3G) induced significant activation. The effect of M3G was significantly stronger than that induced by an equimolar concentration of morphine, whereas the opioid receptor-active metabolite, morphine-6-glucuronide (M6G), did not show any activation potential. Several opioid analgesics were also examined, including (+)-methadone, (−)-methadone, levorphanol, pethidine, buprenorphine, fentanyl, and oxycodone (10–100 µM); all were found to produce significant TLR4 activation in HEK293 cells. The opioid receptor antagonists (−)-naloxone and (−)-naltrexone and their stereoisomers did not, however, display any TLR4 activation potential in HEK293 cells. In addition, (+)-naloxone inhibited morphine and M3G TLR4 signalling effects in a dose-dependent manner [39].

A subsequent study examined the effects of opioid agonists on LPS-induced TLR4 activation in HEK-Blue™ hTLR4 cells, with a view of characterising the pharmacological nature of the interaction [40]. Morphine (3 and 10 µM) and fentanyl (0.3 µM) were found to induce minor, but significant, increases in the activation of TLR4, compared with unstimulated cells. In contrast, morphine and fentanyl significantly inhibited the LPS-induced TLR4 activation in HEK-Blue™ hTLR4 cells. Treatment of cells with 100 ng/mL LPS in the presence of morphine (3–100 µM) resulted in significantly less TLR4 activation when compared with cells stimulated with LPS alone. Similarly, fentanyl (1–100 µM) significantly inhibited LPS (30 ng/mL)-induced TLR4 activation. Moreover, the opioid antagonists, naltrexone (30–1000 µM) and β-funaltrexamine (β-FNA) (3–30 µM), did not activate TLR4 on their own, but significantly inhibited LPS-induced TLR4 activation, as previously reported. Further adding to the confusion, Skolnick et al. attempted to replicate the work of other groups [38,66] that reported on the blockade of TLR4 signalling by opioid antagonists, but were unable to replicate the findings [42]. It has been proposed that the HEK-Blue™ hTLR4 assay fails to include the binding proteins that promote the binding of ligands to the TLR4 complex, which could contribute to the discrepancy [67] and highlight the poor translation of in vitro models to in vivo effects.

Studies reporting the effects of different opioid agents on TLR4 signalling are summarised in Table 1 and Table 2. These studies, while mostly presenting accumulating evidence on the effects of opioids via TLR4 signalling, reveal some discrepancies regarding the mode of action exerted by different opioid agents (agonists vs. antagonists). Several studies indicate that opioid receptor agonists and antagonists exert their effects at TLR4, by activating or blocking TLR4 activation, respectively, in a non-stereoselective manner. However, other studies report that opioid receptor agonists also have the ability to antagonise LPS-induced TLR4 activation. Studies conducted in our laboratory confirm some, but not all, of the above-mentioned results. We have observed that M3G can weakly but consistently activate TLR4 signalling, and that M3G and morphine both significantly inhibit LPS-induced activation [49]. It is worth noting that, rather than a direct interaction at TLR4, some of the literature invokes cross-talk between TLR4 and OR signalling pathways, including cross-targets such as p38 MAPK [27], PKCβ2 [53], or NF-κB [55]. This will be discussed in a later section.

## 5. Opioids Interact with TLR4 through its Accessory Protein (MD-2)

Many studies have utilised in silico molecular docking techniques to predict the interactions between opioids and TLR4. The structures of opioids, including morphine [39,72,73,74,75], naloxone [39,68,72,74,76], naltrexone [39,77], and methadone [39,74], as well as the morphine metabolites, M3G [39,68,72,73] and M6G [39], have all been docked into the crystal structure of TLR4 (PDB ID 3FXI) [78]. This crystal structure contains the symmetrical heterodimer of TLR4 and MD-2. Interestingly, in all the studies that included MD-2 in the docking procedure, the opioids preferentially docked into the LPS-binding pocket of the MD-2 protein [39,68,72,73,74,75,77].

As might be anticipated for the LPS-binding pocket, the majority of opioid–MD-2 interactions predicted by docking studies are hydrophobic interactions [72,77]. The docking of (−)-morphine, M3G, (+)-methadone, and (+)-naloxone within the pocket resulted in overlapping binding poses, suggesting competitive binding [68,74,75]. Interestingly, while opioid–MD-2 docking is non-stereoselective, with both the (+) and (−) stereoisomers predicted to interact favourably [39], the specific binding poses of the stereoisomers differ. (+)-Morphine, for example, is predicted to bind in a way that does not overlap with (+)-naloxone [74], unlike (−)-morphine, which docks with a similar pose to (+)-naloxone [75]. Despite the evidence for competitive binding based on overlapping docking poses, when (−)-morphine, M3G, (+)-methadone, and (+)-morphine were docked into the (+)-naloxone–MD-2 complex, altered binding poses were predicted, suggesting non-competitive binding is also possible [74,75].

The predicted binding pose, and the binding energies of the opioids, may also change depending on the state of the MD-2 into which the opioids are being docked. M3G and remifentanil were both predicted to bind more strongly in the TLR4/MD-2 dimer, compared with MD-2 alone [73,75]. M3G docked even more strongly with the TLR4/MD-2 heterodimer, and resulted in a different binding pose between the two TLR4 proteins [73]. Therefore, it was suggested that the activation of TLR4/MD-2 guides M3G down an energy gradient to its final binding site. The predicted binding energy of (−)-morphine was unaffected by the conformational state of TLR4/MD-2 [73,75]; however, docking into the TLR4/MD-2 heterodimer resulted in a similar binding pose as that of M3G [73].

These studies only examined the interactions of opioids with TLR4/MD-2 using static docking methods; however, more thorough investigations were conducted utilising molecular dynamics simulations to model the changes in opioid binding and protein conformations over time [69,72,77]. In these studies, hydrophobic interactions were emphasised as the most important factor for opioid binding within the LPS-binding pocket, causing the MD-2 structure to “clamshell” to accommodate the opioids [72,77]. Over the course of molecular dynamics simulations, morphine that docked solely into MD-2 lost all its interactions, while morphine docked with the TLR4/MD-2 heterodimer retained stability throughout the simulation [69,72]. This was due to the hydrophobic interactions of morphine with residues of the “Phe126 loop”, also known as the gating loop. This loop encloses the solvent-accessible region of the LPS-binding pocket and controls the activation of MD-2 through the conformation of the Phe126 amino acid. The residues of the “Phe126 loop” undergo large orientation shifts during molecular dynamics of MD-2 alone, explaining the instability of the predicted morphine binding. However, the presence of TLR4 facilitates electrostatic and hydrogen bonds between residues of the “Phe126 loop” and TLR4, stabilising the region and, consequently, the binding of morphine. This predicted binding did not change the conformation of Phe126, retaining the active state of MD-2 [72]. M3G was predicted to overlap with the scaffold of morphine and direct the glucuronide moiety to hydrogen bond with residues that are deeper within a solvent-inaccessible region of the pocket. This binding was stable across molecular dynamics simulations, irrelevant of the presence of TLR4, and did not alter the active state of MD-2. Meanwhile, naloxone acted similarly to morphine in molecular dynamics, requiring TLR4 to maintain stable binding; however, this binding caused Phe126 to change conformation, inactivating MD-2.

In conclusion, the in silico studies indicate that opioids interact with TLR4 primarily by binding in the LPS-binding pocket of MD-2. This binding is non-stereoselective, is governed by hydrophobic interactions, and varies depending on whether MD-2 is separate from, or part of a TLR4/MD-2 heterodimer. Different opioids are predicted to bind in multiple poses throughout the LPS-binding pocket and may or may not directly affect the activation state of MD-2 through the “Phe126 loop”. This suggests the possibility of both competitive and non-competitive binding with the protein.

## 6. The Effect of Opioids on TLR4 Is Non-Competitive

Pharmacological examination of the mechanism of the action of opioids on LPS-induced TLR4 activation indicates a non-competitive antagonism. This was inferred by comparison of the effect of opioids with those of the competitive antagonist LPS-RS on the full LPS concentration-response curve in HEK-Blue™ hTLR4 cells. LPS induced a concentration-dependent increase in TLR4 signalling. Cotreatment with 10 ng/mL LPS-RS did not change the LPS E_max_ value, but caused a parallel, rightwards shift of the curve, significantly increasing the EC_50_ value from 0.85 to 2.16 ng/mL. Conversely, cotreatment with either fentanyl or the opioid antagonist β-FNA reduced the E_max_ values and caused a non-parallel, rightwards shift of the LPS response curve to the right (increased EC_50_) and downwards (decreased E_max_), which suggested a low capacity binding site or a non-competitive antagonism [40].

## 7. Opioids Affect NF-κB Activation, Downstream of Both TLR4 and Opioid Receptors

NF-κB is a major downstream signalling element in TLR4-mediated inflammatory pathways [79], and the effects of opioids on LPS-induced NF-κB activation have been evaluated. Opioid receptor gene ablation studies have shown that opioids activate or downregulate NF-κB signalling in different cell types, resulting in the modulation of immune and neuronal responses (reviewed by [80]). The modulatory effects of morphine, particularly on LPS-induced NF-κB activation, were examined in mouse and human immune cells [81]. In mouse peritoneal macrophages, pre-treatment with nanomolar morphine concentrations (50 nM) for 2 h increased LPS-induced NF-κB activation, as well as IL-6 and TNF-α secretion and mRNA levels; these effects were reversible through adding naloxone. Conversely, morphine micromolar concentrations (50 µM) inhibited LPS-induced IL-6 and TNF-α secretion and reduced NF-κB activation; however, these latter effects were not reversed upon adding naloxone. Further supporting differential mechanisms for the effects of different morphine concentrations on LPS-induced NF-κB activation, the transfection of primary microglial cells with siRNAs that target the expression of µ-opioid receptor blocked the potentiating effect of a low concentration of morphine (100 nM) on LPS-induced NF-κB activation, while only reducing the effect of high morphine concentrations (10 µM) [45]. These results indicated MOR-mediated effects for low concentrations of morphine, but MOR-independent effects for high concentrations of morphine. In contrast, while morphine alone did not induce any activation, morphine pre-treatment resulted in a concentration-dependent, naloxone-sensitive inhibitory effect on LPS-induced NF-κB nuclear translocation [82]. The underlying mechanism was suggested to be a capability of morphine to induce nitric oxide (NO) release, as the morphine inhibitory effect was entirely blocked by the NO synthase inhibitors N_ω_-nitro-_L_-arginine-methyl-ester and N_ω_-nitro-_L_-arginine.

The ability to modulate LPS-induced NF-κB activation was also reported for opioid peptides. The effects of the opioid peptides endomorphins 1 and 2 on human THP-1 cells differentiated into macrophage-like cells was evaluated [83]. Both peptides (10^−8^ and 10^−6^ M) augmented NF-κB nuclear translocation independently; furthermore, they significantly potentiated LPS (1 µg/m)-induced activation in a concentration-dependent fashion. However, neither of the two opioid peptides had an influence on the production of NF-κB targets IL-10 and IL-12, and they significantly mitigated their LPS-induced production in a concentration-dependent manner. The authors propose that endomorphins may induce the translocation of NF-κB homo- and hetero-dimers that are different from those translocated upon stimulation by LPS. Further studies have elaborated on the interplay between MOR and TLR4 activation by opioids, demonstrating that they have opposing impacts on NFκB activation [73].

Taken together, the data illustrate that the effects of opioids on LPS-induced activation vary between potentiation and inhibition when different opioid concentrations and different cell types are examined. While LPS induces NF-κB activation through TLR4, the actions of opioids seem to encompass more complexity, involving simultaneous activity at both opioid receptors and TLR4. This has been reported as a cross-talk between the two signalling pathways [84].

## 8. Opioids Modulate TLR4 Expression

Changes in the expression levels of TLR4 upon opioid treatment have been reported in several in vitro studies. The expression of TLR4 mRNA was up-regulated by morphine exposure (200 µM) in microglial BV-2 cells [85]. An increase in TLR4 protein expression in the same cell line was detected at a lower morphine concentration (10 μM) [86]. Treatment of lumbar dorsal spinal cord tissue with 100 µM morphine also significantly increased TLR4 protein expression; whereas, the respective 10 µM concentration had no effect on TLR4 protein levels [87]. In human CHME-5 microglia, morphine (10 µM) induced up-regulation in the expression of TLR4 protein in the presence of LPS. Methadone alone up-regulated TLR4 protein expression but had the opposite effect when combined with LPS, as did oxycodone or buprenorphine [88]. Similarly, endomorphin-1 inhibited the expression of TLR4 on peripheral blood dendritic cells that were stimulated by high glucose [89].

Similar effects have been reported for various opioids in several in vivo studies, employing various dosing regimens. Chronic intrathecal administration of morphine, (−)-methadone, or (+)-methadone to rats (15 µg, once daily for a week) induced spinal glial activation, as well as significant elevations in mRNA and in protein levels of TLR4 in the lumbar dorsal spinal cord [74,87]. Another study by the same group also reported spinal glial activation and elevation in TLR4 mRNA levels, following acute intrathecal administration of 0.75 μg M3G to rats [68]. Morphine exposure in vivo can induce long-term changes in TLR4 expression by microglial cells; rats exposed to morphine during adolescence have increased expressions of TLR4 mRNA and protein in the microglia of the nucleus accumbens during their adulthood [90]. Epithelial cells, isolated from the small intestines of mice implanted with 75 mg morphine pellets for 24 h, demonstrated up-regulated expression of TLR2, TLR4 mRNA, and protein levels [91]. In contrast, remifentanil preconditioning inhibited TLR4 expression in liver tissue in a mouse model of hepatic ischemia reperfusion injury [92] and the κ-opioid receptor agonist U50, 488H mitigated the ischemia/reperfusion-induced myocardial TLR4 expression [93]. Overall, the in vitro and in vivo literature indicate that opioid administration per se increases TLR4 expression on a variety of cell types of both central and peripheral relevance, while in pro-inflammatory conditions opioids prevent TLR4 induction.

Pro-inflammatory responses and glial activation following peripheral and spinal nerve injury are associated with up-regulation in the expression of spinal TLR4 mRNA [94,95,96]. The up-regulation of TLR4 mRNA, induced by SNAP (Spinal Neuropathic Avulsion Pain) surgery, was shown to be further amplified upon morphine administration [97]. Subcutaneous morphine administration for one week (10 mg/kg once daily in Sprague-Dawley rats after surgery) potentiated the SNAP-induced elevation of TLR4 mRNA and pro-inflammatory cytokines in the ipsilateral dorsal spinal cord tissue. Similarly, spinal TLR4 mRNA levels were increased following a week-long subcutaneous morphine treatment in mice with tibia fractures [98].

In contrast to these results, morphine treatment decreased TLR4 mRNA and protein levels in RAW 264.7 cells in vitro, as well as in peritoneal macrophages isolated from morphine-treated BALB/cJ mice; whereas, TLR4 mRNA and protein levels were increased upon naloxone treatment in both cases [99]. In addition to expression levels, it is possible that opioids modulate the subcellular localisation of TLR4. Experiments using the endocytosis inhibitor dynasore suggested that morphine treatment decreases the surface expression of TLR4 in microglia by causing increased TLR4 endocytosis [70].

## 9. Functional Consequences of TLR4 Activation by Opioids

Depending on the cell type affected, and the localisation (central or peripheral) of the interaction, TLR4 activation by opioids is proposed to cause neuro-inflammation [69,73], leading to opioid-induced hyperalgesia [39,100]; play a role in dependence, reward, and reinforcement [75,101]; and contribute to morphine-induced suppression of colon peristalsis [102,103].

### 9.1. Opioid Receptor-Independent Mechanisms Contribute to Opioids Deleterious Effects

Some of the earliest evidence suggesting a non-GPCR activity of opioids came from a series of studies that showed that the antinociceptive effect induced by morphine, in male CD-1 mice, was supressed upon intrathecal pre-treatment with the opioid-active (−)-morphine as well as the opioid-inactive (+)-morphine. The anti-analgesic effects of both morphine isomers were blocked upon pre-treatment with (+)-naloxone, further confirming the involvement of a non-opioid receptor-mediated mechanism. Moreover, (−)-morphine and (+)-morphine mitigated the antinociceptive effect of the δ-opioid receptor agonist deltorphin II, and the κ-opioid receptor agonist U50, 488H, in μ-opioid receptor knockout mice [104,105,106]. LPS was reported to reduce morphine analgesia in rats through several contributing mechanisms [107,108]. The effect of LPS was suppressed upon pre-treatment with the non-competitive NMDA receptor antagonist (MK-801), the glial metabolic inhibitor (fluorocitrate), and the opioid receptor antagonist (naloxone). In another study, the antinociceptive effect of morphine in male CD-1 mice was attenuated in a dose-dependent fashion upon pre-treatment with LPS (1.5–4 µg). However, this effect of LPS was reversed upon pre-treatment with both levo-naloxone and its opioid receptor-inactive isomer dextro-naloxone, denoting the involvement of a non-opioid mechanism [109].

Evidence suggesting the contribution of CNS glial cells to pain behaviours was first reported 25 years ago [110] and, subsequently, multiple studies emerged investigating the mechanisms mediating the development of neuropathic pain via glial activation [111,112]. Later, opioid-induced pro-inflammatory glial activation was shown to be responsible for the adverse effects of opioids, which include the opposition of analgesia, analgesic tolerance, hyperalgesia, respiratory depression, opioid dependence, and opioid reward. It was shown that drugs which suppress glial activation, and subsequent cytokine production, reinforce the antinociceptive effect of morphine and reverse neuropathic pain, tolerance, and hyperalgesia [113,114,115,116]. However, the mechanistic basis of these opioid pro-inflammatory effects was unclear, until the discovery of opioid activity at TLR4. This formed the basis for multiple studies employing a range of in vivo pharmacological and genetic manipulations to investigate the TLR4-mediated effects of opioids.

### 9.2. Central and Peripheral Neuropathic Pain

TLR4 is expressed in the central nervous system on microglia, astrocytes, and endothelial cells [117]. Sensory neuronal damage initiates several neuron-to-glia activation signals, one of which is through the activation of TLR4, as expressed on glial cells by endogenous “danger” signals released upon nerve injury [118,119]. The role of TLR4 in neuroimmune activation following nerve injury was demonstrated in animal models of neuropathy. A significant reduction in the expression of spinal microglial activation markers and pro-inflammatory cytokines, together with significant attenuation of behavioural hypersensitivity, were observed in TLR4 knockout and point mutant mice, and also upon intrathecal administration of TLR4 antisense oligodeoxynucleotide to rats [120].

Based on recent in vitro data that has established the TLR4-antagonistic effects of the neuronally inactive (+)-naloxone and (+)-naltrexone [38,48], their influence on neuropathic pain was tested using a model of peripheral neuropathy, via partial sciatic nerve chronic constriction injury. A significant attenuation of mechanical allodynia was observed after intrathecal administration of (+)-naloxone or (+)-naltrexone (60 μg), as well as following subcutaneous administration of (+)-naloxone (100 mg/kg). Moreover, the sustained delivery of (+)-naloxone or (−)-naloxone via intrathecal infusion (60 μg/h, 4 days) completely reversed the established neuropathic pain [38]. The TLR4-mediated effects of opioids were also explored in models of central neuropathy, where (+)-naloxone was reported to reverse mechanical allodynia resulting from spinal cord injury [121]. Moreover, the subcutaneous administration of morphine after spinal injury caused a significant elevation of mechanical allodynia, and this effect was blocked by co-administration of (+)-naloxone [97].

### 9.3. Analgesia, Hyperalgesia, Tolerance, and Dependence

TLR4 signalling may be involved in opposing acute opioid analgesia, and in the development of tolerance, hyperalgesia, and dependence [38]. Pharmacological blocking of TLR4 activation and its downstream signalling on the analgesic effects of morphine were evaluated. The evaluation demonstrated a significant potentiation of the magnitude and duration of morphine analgesia upon co-administration of the competitive TLR4 antagonist LPS-RS, or of a Toll-Interleukin-1 receptor domain, containing adaptor protein (TIRAP) inhibitor peptide. It was also reported that (+)-naloxone significantly increased systemic and intrathecal morphine analgesia and alleviated the effects of chronic morphine administration, including tolerance, hyperalgesia, and dependence.

In earlier studies, M3G was reported to cause pain enhancement and induce allodynia and hyperalgesia, since, however, M3G lacks activity at all opioid receptors, the mechanism involved remains unknown [122]. Based on subsequent in vitro cell studies that reported TLR4 activation by M3G, the triggering of a pro-inflammatory response by the TLR4-mediated activation of immune cells emerged as a possible mechanism underlying the pain-enhancing effects of M3G. Intrathecal administration of M3G (0.75 μg) to rats induced potent mechanical allodynia and thermal hyperalgesia, which were both blocked upon co-administering pro-inflammatory cytokine and glial inhibitors, and also upon co-administering either isomer of naloxone [68].

While most studies evaluating the non-neuronal effects of opioids focused on microglia and astrocytes, one study attempted to determine whether opioids may have a TLR4-mediated effect on CNS endothelial cells [73]. After confirming the expression of functional TLR4 by primary adult rat CNS endothelial cells, the effects of intrathecal injections of opioid-stimulated endothelial cells on in vivo behavioural responses were evaluated. Cells treated in vitro with M3G induced significant tactile allodynia in rats compared with saline-treated cells; whereas, cells stimulated with (−)-morphine, or co-treated with M3G and LPS-RS, did not have any significant effect.

Morphine tolerance resulting from chronic subcutaneous administration was shown to be associated with an increase in glial cell activity in the ventrolateral periaqueductal gray (vlPAG) [123]. Through subsequent pharmacological studies, it was demonstrated that TLR4 signalling in the vlPAG glia is involved in developing opioid tolerance. Intra-vlPAG microinjections of both LPS-RS and (+)-naloxone prevented the development of tolerance to systemic morphine, whereas intra-vlPAG microinjections of LPS or (+)-morphine were shown to induce naive tolerance [124].

### 9.4. Effect of Pharmacological or Genetic TLR4 Ablation on Algesia, Reinforcement, Tolerance, and Dependence

Hyperalgesia can be triggered by the continuous infusion, for several days, of morphine or oxymorphone to the opioid receptor of triple knockout mice; this suggests the involvement of an opioid receptor-independent mechanism [125]. Several genetic manipulation studies point to the involvement of TLR4 in mediating the effects of opioids. The dose–response curve for acute morphine analgesia (1–50 mg/kg) demonstrated a significantly higher analgesic effect for morphine in TLR4 knockout and MyD88 knockout Balb/c mice when compared with their wild-type control [39,69]. Co-administration of (+)-naloxone (60 mg/kg) significantly increased the analgesic effect of morphine (2.5 mg/kg) in wild-type mice, while having no influence in TLR4 knockout mice [39]. A study tested the effects of two small-molecule inhibitor compounds that reportedly targeted TLR4 and its accessory protein MD-2, causing disruption of the TLR4/MD-2 complex [69]. Both compounds potentiated the acute analgesic effects of morphine in rats; whereas, in Balb/c mice, the potentiating effect was only attained in the wild-type strain and not in the TLR4 knockout strain. The acute analgesic effect of oxycodone was also stronger in the absence of TLR4, where significantly longer hot plate latencies were recorded over a range of oxycodone doses (0.01–5 mg/kg, I.P.) in Balb/c TLR4^−/−^ mice vs. wild-type mice [75]. Conversely, in a subsequent study by the same group, acute morphine analgesia produced by a single dose of morphine (7.5 mg/kg) in the same mouse strains was found to be similar in wild-type, TLR4 knockout, and MyD88 knockout mice [126]. It is worth mentioning that several factors may contribute to these inconsistent responses, including the behavioural test and assay protocol used, as well as the mouse strains employed. The opioid dose and its magnitude of response could also have an impact, where the differences between analgesic effects may be masked at doses approaching minimum or maximal analgesia.

The detrimental effects of opioids were also shown to diminish in TLR4 mutant and knockout mice. Repetitive injections of escalating doses of morphine to wild-type Balb/c and MyD88 knockout mice over 3 successive days resulted in significant loss of analgesia and the development of tolerance. However, analgesic tolerance was not observed in TLR4 knockout mice, implying the involvement of a MyD88-independent TLR4-mediated mechanism [126]. Intraperitoneal M3G (25 mg/kg) induced tactile hyperalgesia only in wild-type (C57BL/6) mice; whereas, hyperalgesia was significantly abolished in TLR4 mutant mice (C57BL/10ScNJ) [127]. Thermal hyperalgesia and mechanical allodynia, resulting from repeated intraperitoneal administration of codeine (21 mg/kg) or morphine (20 mg/kg) to wild-type Balb/c mice, were not observed in TLR4 null mice [128]. TLR4 ablation also protected against mechanical hyperalgesia that was induced by subcutaneous infusion of remifentanil (4 mg/kg/min, 1 h, for 3 days) [100]. The behavioural reinforcing actions of oxycodone, assessed by conditioned place preference tests, were significantly reduced in TLR4 knockout and MyD88 knockout Balb/c mice compared with the wild-type control [75], suggesting the involvement of TLR4 signalling in opioid reinforcement and reward effects.

Interestingly, studies have also presented evidence that supports the absence of a role for TLR4 signalling in some deleterious opioid actions. No difference was found between the extent of tactile hyperalgesia induced by morphine in TLR4 mutant mice (C3H/HeJ) and that observed in their wild-type controls (C3H/HeOuJ) [129]. Chronic morphine administration (60 mg/kg, once daily for 5 days) was also reported to induce analgesic tolerance to the same extent in TLR4 mutant mice (C3H/HeJ) when compared to the wild-type (C3H/HeN), as well as in TLR4 knockout C57BL/6 mice compared to wild-type. Moreover, the mRNA expression of the microglial activation marker CD11b was significantly increased after chronic morphine administration in knockout and wild-type mice, suggesting that TLR4 is not involved in opioid–microglial activation [130]. In another study, two mouse strains were employed to test opioid effects: TLR4 mutant (C3H/HeJ) vs. wild-type (C3H/HeOuJ), and TLR4 null (B10ScNJ) vs. wild-type (B10ScSNJ). All morphine effects including antinociception, hyperalgesia, tolerance, and physical dependence were unchanged in TLR4 mutant and TLR4-null mice [131]. The discrepancies in the effect of TLR4 ablation on morphine pharmacodynamics have been proposed to be due to confounders in studies, e.g., higher morphine-induced analgesia in TLR4-knockout animals compared with the control strain, the effect of opioid binding to MD2 in absence of TLR4, and the concentration of opioids to which receptors are exposed in vivo [130,131].

### 9.5. GIT and Colon Motility

It is well recognised that one of the major undesirable effects of opioid analgesics is their gastrointestinal side effects, which are perceived as a key limitation to their therapeutic utility [132,133]. Toll-like receptors, including TLR4, are widely expressed in the GIT and have been linked to the development of GIT immune responses and to several gastrointestinal pathologies [134]. Owing to the observations of opioid interactions with TLR4 in the nervous system, the role of toll-like receptors in mediating opioid GIT effects have been investigated. One of the mechanisms implicated in the disruption of intestinal tight junctions is through the activation of toll-like receptors [135,136]. Accordingly, TLR involvement in mediating the effects of morphine on the intestinal barrier function was investigated. Morphine-induced gut bacterial translocation to the mesenteric lymph node and liver was completely abolished in μ-opioid receptor knockout mice and was significantly mitigated in TLR4^−/−^, TLR2^−/−^, and TLR2/4^−/−^ double knockout mice [91]. Morphine was shown to disrupt the tight junction protein organisation between intestinal epithelial cells. This effect was significantly attenuated in TLR4^−/−^ mice and completely abolished in mice lacking μ-opioid receptor, and in TLR2^−/−^ and TLR2/4^−/−^ double knockout mice, which implies that disruption of intestinal barrier function by morphine is partially mediated by TLR4.

Opioids are well-known for their inhibitory effects on the gastrointestinal motility, and opioid-induced constipation is a serious limitation of opioid therapy [137,138]. TLR4 activation by opioids is proposed to contribute to the morphine-induced suppression of colon peristalsis [102,103]. Pre-treatment with the TLR4 antagonist TAK-242 significantly alleviated the morphine-induced inhibition of colon peristalsis and propulsion velocity in the isolated guinea pig colon in vitro and in mice in vivo [102]. In a more recent study, morphine’s inhibitory effects on the gastrointestinal transit in wild-type BALB/c mice were shown to be significantly attenuated in TLR4^−/−^, TLR2/4^−/−^ and MyD88^−/−^ knockout mice, where a subcutaneous injection of morphine (10 mg/kg) was shown to retard the movement of ingested content along the GIT in wild-type mice. However, this differential effect was not replicated in vitro, where no differences were observed between the responses to morphine for wild-type and TLR2/4^−/−^ isolated colon preparations, suggesting the involvement of a pathway extrinsic to the colon [103]. Taken cumulatively, these data suggest that morphine may exert its effects on the GIT through acting directly on toll-like receptors or through a mechanism involving cross-talk between μ-opioid and toll-like receptor signalling.

## 10. Whether TLR4 Mediate the Effects of Opioids on Tumour Growth and Metastasis Is Unexplored

It is quite remarkable that, despite a well-documented link between TLR4 and cancer (reviewed in [7,139,140]), and reasonably convincing evidence that opioids are active at TLR4, the possibility that TLR4 may mediate the effects of opioids on tumour growth and metastasis has not, to date, been explored.

From a clinical perspective, opioids will remain the mainstay analgesics in patients with cancer, although they have been scrutinized for negatively affecting tumour biology. Opium has been implicated in cancer development [141] most likely due to mutagenic compounds induced by pyrolysis. Chronic use of prescription opioids has been associated with a higher morbidity and overall mortality; however, evidence for increased carcinogenesis in these patients is lacking [142]. With respect to the perioperative use of opioids in cancer surgery, the most recent meta-analysis by Zheng et al. in a mixed cancer population indicated that neither overall survival nor progression-free survival was affected by the intraoperative use of opioids [143]. For those patients with advanced cancer, higher opioid doses are usually required to effectively treat pain.

Pain itself is a major contributor impairing host resistance and promoting tumour progression; thus, there has been advocation for adequate pain treatment to be tumour-protective [144]. Differentiation between the suggested “tumour-protective” action of opioids—due to their effective analgesic properties—and their hypothesized immune-related “tumour-promoting” effects, regarding clinical relevance in oncological patients, is challenging. It is thus not surprising that, in a recently published large scaled trial including 2132 patients with breast cancer, the use of regional anaesthesia—which decreases opioid administration—did no better than perioperative use of opioids, with regard to local or metastatic breast cancer recurrence [145].

Recognition that some opioids weakly activate TLR4, but significantly prevent TLR4 activation induced by agonists, needs to be added to the list of factors that may contribute to the complexity and existing discrepancy in the field of opioid influence on cancer. We have demonstrated that opioid activity of perioperative plasma samples correlates with inhibition of TLR4 activation [65]. Interestingly, using linear mixed models, this study also found that the ability of plasma samples to activate TLR4 has a significant effect that explains pain scores [65]. Therefore, the interrelation between pain, TLR4 activation, and opioids is anticipated to be an important avenue of research for basic scientists and clinicians.

## 11. Conclusions

The discovery of opioid activity at TLR4 has allowed us to explain a number of OR-independent effects for this class of drugs and forced a number of paradigms to evolve in opioid pharmacology. The discovery also suggests the possibility that a number of undesirable effects of opioids may be mitigated by the development of pharmacotherapies targeting TLR4, to enhance the safety and efficacy of opioids. Furthermore, research in the near future will likely attempt to identify whether opioids interfere with the activation of TLR4 by the endogenous molecular patterns (DAMPs) that are relevant to a number of pathologies in which patients are likely to be administered opioids.

## Figures and Tables

**Table 2:** In vitro studies testing TLR4 activation by various opioids.

Cells	Opioid Agent	Concentration	TLR4 Activation Readout	Effect of Opioid Agent	Reference
RAW264.7 murine macrophages	(+) and (−)-Morphine	200 µM	GFP-Akt1 cytosolic clearance and the effect of LPS-RS	Significant activation, inhibited by LPS-RS or (+)/(−)-Naloxone	[39]
HEK-Blue™ hTLR4 cells	(+) and (−)-Morphine (+) and (−)-Methadone M3G Levorphanol Pethidine Buprenorphine Fentanyl Oxycodone M6G (+) and (−)-Naloxone (+) and (−)-Naltrexone (+)-Nalmefene	10 µM 10 µM	Increase in SEAP expression	Significant activation No activation	
HEK-Blue™ hTLR4 cells	M3G	10 µM	Increase in SEAP expression	Significant activation, dose-dependently suppressed by LPS-RS	[68]
Primary adult rat CNS endothelial cells	(+)-Morphine	50 µM 100 µM	Phosphorylation of MAP kinases (p38 and ERK)	Increase in p38 phosphorylation Increase in p38 and ERK phosphorylation	[69]
Primary adult rat CNS endothelial cells	(+)-Morphine	100 µM	mRNA expression of IL-1β, TLR4, and MD-2 and the effect of coincubation with LPS-RS or the intracellular TLR4 antagonist CLI-095	Increase in mRNA expression of IL-1β, TLR4, and MD-2, significantly attenuated by LPS-RS Increase in mRNA expression of TLR4 and MD-2, significantly attenuated by CLI-095	
BV-2 dual luciferase NF-κB reporter cells	Morphine	25–400 µM	NF-κB activity (Dual-Glo luciferase assay) and the effect of coincubation with the MD-2 competitive inhibitor curcumin	Concentraton-dependent activation of NF-κB, suppressed by curcumin in a concentration-dependent manner	
HEK-Blue™ hTLR4 cells	Morphine	400 µM	Increase in SEAP expression	Significant activation, inhibited by LPS-RS in a concentration-dependent manner	
BV-2 microglia cells	Morphine	200 µM	Protein levels of IκBα and NF-κB p65 and the effect of coincubation with the MD-2 competitive inhibitor curcumin	Induced IκBα-degradation, significantly inhibited by curcumin Increased NF-κB p65-protein expression, significantly inhibited by curcumin	
BV-2 microglia cells	Morphine	400 µM 200 µM	NO production and IL-1β and TNF-α protein levels and the effect of RNAi knockdown of TLR4 or MD-2	Induced NO production, suppressed by TLR4 and MD-2 RNAi knockdown Increased IL-1β and TNF-α expression, inhibited by TLR4 and MD-2 RNAi knockdown	
HEK-Blue™ hTLR4 cells	Morphine Fentanyl Naltrexone β-FNA	3 and 10 µM 0.3 µM 3–1000 µM 3–30 µM	Increase in SEAP expression	Minor significant activation Not conc. Dependent No activation	[40]
Microglia primary culture	Morphine	100 µM	IL-1β mRNA expression and protein synthesis, and the effect of LPS-RS	Significant activation, inhibited by LPS-RS	[70]
HEK-Blue™ hTLR4 cells	M3G Morphine M6G	0.5–100 µM 0.5–100 µM 0.5–100 µM	Increase in SEAP expression and the effect of LPS-RS	Significant activation, inhibited by LPS-RS No activation (significant only at 10 µM) No activation	[49]
PC12 cells	M3G	30 μM	Expression of polysialic acid or TNFα, cell migration	Increase	[71]

## Data Availability

No new data were created or analyzed in this study. Data sharing is not applicable to this article.
